# Consequences of infertility in developing countries: results of a questionnaire and interview survey in the South of Vietnam

**DOI:** 10.1186/1479-5876-4-54

**Published:** 2006-12-27

**Authors:** Nicole J Wiersema, Anouck J Drukker, Mai Ba Tien Dung, Giang Huynh Nhu, Nguyen Thanh Nhu, Cornelis B Lambalk

**Affiliations:** 1 Division of Reproductive Medicine, Department of Obstetrics and Gynaecology Vrije Universiteit Medical Centre (VUmc), Amsterdam, The Netherlands; 2 Andrology Unit of Binh Dan Hospital, Ho Chi Minh City, Vietnam; 3 Department of Obstetrics and Gynaecology of Tu Du Hospital, Ho Chi Minh City, Vietnam

## Abstract

**Background:**

This study explores the psychological, socio-cultural and economic consequences of infertility on couples' life. The purpose of this research is to improve knowledge about the potentially serious implications of infertility in the South of Vietnam.

**Methods:**

This study included 118 infertile couples who filled in questionnaires and 28 men and women who were interviewed.

**Results:**

Data of the questionnaire show men and women do not differ in their responses and attitudes towards infertility. Almost one-third of the participants require psychological support. Interviewees experience secrecy, social pressure and economic hardship.

**Conclusion:**

Offspring are very important to Vietnamese couples. Their future depends on children. Family plays an important role in the experiences of the infertile couple. Economic consequences are a particular distressing factor. There is a need for psychological counselling in the treatment of infertile couples in the South of Vietnam. It should be realised that in developing countries, despite overpopulation, unwanted childlessness is an important social and economical burden that needs attention.

## Background

Infertility is a problem of global proportions. The WHO estimates that 8–12% of couples around the world experience difficulty conceiving a child [1]. The infertility rates vary between countries and regions. Due to the overpopulation problem in developing countries, overfertility rather than infertility has been the focus of family planning programmes. The consequences of infertility in these countries range from economic hardship, to social isolation, violence and denial of proper death rites. Many families depend on children for economic survival, especially in old age [2]. In oriental cultures reproduction is one of the highest valued factors [3]. A psychological crisis may occur when reproduction appears impossible. The way in which people deal with infertility is at least partly affected by the values and socio-cultural norms of the community in which they live.

Most researchers conclude that infertility is a more stressful experience for women than it is for men. Few studies have explored the effect of a gender-specific infertility diagnosis on the responses of couples. Previous studies reported more negative feelings about infertility and more psychiatric distress among men with male factor infertility compared to men in couples receiving other diagnoses [4-6]. A study in Taiwan comparing the differences in responses from husbands and wives based on an infertility diagnosis, reported that husbands, regardless of the diagnosis, showed no differences in psychological responses. Only wives with diagnosed female infertility expressed higher distress to infertility than their husbands, and wives experiencing a diagnosed male infertility [7].

Studies conducted in Northern Vietnam examined the motives of women for having children. To Vietnamese people, family is the most important unit and is considered as a mini-commune. For women childbearing is associated with stabilizing their marriage and closer bonds with his family [8,9].

Especially eldest daughters-in-law are eager to have their first child very soon, one year after marriage, to demonstrate their fertility. They feel pressured by their parents-in-law to give them a male grandchild who can carry on the family name. Thus for women childbearing is expected to bring happiness and family harmony [8-10].

According to Vietnamese customs, it is normally the son who takes care of and/or supports their parents until they die. Often this role is handed over to their wives. Many married lives start in the husband's family until the couple is able to build their own house. However, after marriage, the wife's main duty is to care for her parents-in-law. Having no son can be a cause of old people's loneliness.

Because of inadequate pension provisions retired people still have to earn their living, which means that children play a crucial role in supporting them, either financially or practically [10].

The experienced social sufferings of women due to childlessness are difficulties concerning integration into the family-in-law [8,9] and their powerless status in the community without children [9].

Little is known about the consequences of infertility in the South of Vietnam and the impact on men. Besides, infertility, although the infertility rate has increased recently, seems to play a minor role in the health care system because the Vietnamese government mainly concentrates on reducing her population by proclaiming a two-child-policy [11]. This study reports on the psychological, economical and socio-cultural consequences of infertility for couples in the South of Vietnam. It is aimed to improve knowledge about the potentially serious implications of infertility in the South of Vietnam.

In order to understand the experiences of involuntary childlessness in the socio-cultural context in which these experiences occur, qualitative and quantitative data were utilized.

## Materials and methods

### Setting

Data in this study were collected from couples with fertility problems who visited Binh Dan hospital or Tu Du Hospital. Both hospitals are located in Ho Chi Minh City, Vietnam.

Tu Du Hospital is the top-referral Hospital in the field of Obstetrics and Gynaecology in the South of Vietnam. The Infertility clinic of the hospital has more than 20.000 visits, with more than 10.000 infertile couples per year. It also conducted about 3.000 intra-uterine insemination cycles together with 2.000 cycles of IVF and related procedures.

Binh Dan referral hospital is specialised in urology and general surgery. The andrology unit was set up in February 2005 and carries out male assessment. The cooperation of the two hospitals began in February 2002 for the treatment of azoospermic men.

All of the patients have to self-finance their infertility treatment expenses. Tu Du Hospital, as the government hospital, subsides part of the hospital fee for their treatment. The total cost for IVF treatment is roughly USD 3.000.

### Procedure

During the period from July until October 2005 couples with fertility problems who visited Binh Dan or Tu Du for consulting or treatment received a questionnaire and an invitation to participate in an interview. Each couple received a stamped and pre-addressed envelope containing two questionnaires with a covering letter explaining the purpose of this study and requesting the couple to fill in the questionnaire separately. The questionnaires were collected in the hospitals or returned by mail in case the couples were incomplete or lacking time. Depending on time schedules and availability of patients and translator some couples participated in an interview. This means it was a quasi-random sample. Consent to conduct this study was obtained from the Department of Health Services in Ho Chi Minh City.

### Questionnaires

The participants completed the questionnaires which contained questions about socio-demographic background, economic background, reproductive history and the Psychological Evaluation Test (PET) [12].

The PET includes fifteen questions detecting emotional reactions to infertility-related stressors. The responses were assigned four grades with respect to frequency (1 = never/rarely; 2 = sometimes; 3 = often, and 4 = always). A PET-score > 30 points is defined as cut-off point for identifying individuals who require psychological support.

Special attention was paid to 'fear of the future', 'the attitude on assisted reproduction and adoption', and 'the need for children as a security for participants' old age'. The last two items could be answered with 'yes' or 'no' and space was reserved for motivating this answer. 'Fear of the future' was measured in the same way as the questions in the PET.

### Interviews

The interviews were semi-structured and held in each participant's preferred language (English or Vietnamese). Participants were interviewed alone in order to prevent that partners would influence each other and give them the opportunity to speak frankly. All interviews were conducted by two English speaking Dutch medical students and an English speaking Vietnamese doctor.

The interview questions focused on the socio-cultural experiences of infertility and were open-ended. New topics were allowed to be explored as they revealed themselves during the interview.

### Translation

Items originally written in English were translated into Vietnamese by one of the doctors, and examined and corrected by another doctor. Answers to open questions were translated by English speaking Vietnamese Doctors.

The interviews were taped, transcribed and when necessary translated into English by an English speaking Vietnamese doctor.

### Data analyses questionnaires

Two tailed, paired *t*-tests were conducted to determine the similarity in the outcome of the psychological evaluation test (PET) between husbands and wives. One-way analysis of variance (ANOVA) were conducted to compare the effect of different diagnoses on husbands' and wives' responses respectively. A *P*-value less then 0.05 was considered statistically significant.

## Results questionnaires

### Sample

The sample consisted of 118 couples (236 participants), who were patients at Binh Dan or Tu Du Hospital. Men were significantly older (mean ± SD 32.24, 5.4) than their partners (mean ± SD 29.11, 5.1) [t (117) = 10.155, *P *< 0.001]. Male infertility was diagnosed in 35 couples. In 24 couples there was female related infertility. Both male and female factor infertility was diagnosed in 6 couples. The remaining 53 couples were either not yet diagnosed or unexplained infertile. The couples had been married for 4.22 ± 3.5 years. The duration of infertility was often unknown and therefore not calculated. All couples experienced primary infertility. Couples were in different stages of treatment: 19 first consult, 15 under examination, 13 diagnosed (not yet under treatment), 47 under treatment (not IVF or ICSI), 6 IVF and 18 ICSI. None of the couples were pregnant when participating in this study. Sixteen percent of the participants had finished primary school, 23 % finished lower secondary school, 39 % finished upper secondary school, 21 % had education beyond college and 1 % is unknown. Fifty-six percent has an income per month of < USD 140, 19.5 % USD 140–350, 3.4 % USD 350–700, 1.3 % > USD 700 and 19.5 % did not want to answer how much they earn. All couples are from the South of Vietnam, 41.5 % living in Ho Chi Minh City.

### Outcomes

Group assignment (male, female, both, unexplained/not yet diagnosed infertility) was based on self-report data. The cause of infertility was not compared with the medical charts. Forty-seven-and-a-half percent of the couples recognised their fertility problem within 2 years after marriage.

Paired *t*-tests were computed in each group of couples to examine differences in responses between husbands and wives. No significant difference was found between the mean PET-score for husbands (27.38 ± 7.1) and wives (27.92 ± 7.3). The Pet-scores between husbands and wives also did not differ significantly in the assigned groups (Table 1).

**Table 1:** Effect of gender differences on scores of the Psychological Evaluation Test (PET)

	Male infertility (n = 35 couples)			Female infertility (n = 24 couples)			Both infertile (n = 6 couples)			Unknown (n = 53 couples)		
	Husbands	Wives	P-value^a^	Husbands	Wives	P-value^a^	Husbands	Wives	P-value^a^	Husbands	Wives	P-value^a^

**PET**	27.30	27.21	NS	27.65	28.36	NS	27.29	33.40	NS	27.35	27.63	NS

^a ^P-value obtained with two-tailed paired T-test

NS: not significant

A PET-score higher than 30 was found in 30.9% of the participants. In this group of participants there was no significant difference found between husbands and wives in the assigned groups.

The effect of the infertility diagnoses on the responses of infertile husbands and wives was studied with a one-way ANOVA. The ANOVA was computed separately for husbands and wives. The results showed no significant differences. No differences in psychological responses were found among husbands regardless of their infertility-diagnosis. The same can be said regarding the wives.

The question 'do you fear the future without children?' revealed 49 % responses of the high frequency type (score 3 and 4). No significant difference was found between husbands and wives.

The question 'do you consider assisted reproduction?' was answered with yes by 67% of the participants, 5 % had no opinion. Motivations for not considering assisted reproduction were unanimously the high costs of treatments.

Adoption was considered by only 18% of the participants, 6 % had no opinion and the remaining 76 % answered 'no'. Counter-arguments were "I want kids of my own" or "An adopted child is not my biological child". There is no significant difference in the attitude on adoption and assisted reproduction between husbands and wives.

Ninety-five percent sees children as a security for old age. There is no significant difference between men and women in this matter. Dependence on children, consolidation of their future and children taking care of their parents were motivations for this statement.

Twenty percent preferred a son, 2.5 % a daughter and 77.5% did not have a preference. Men and women did not differ significantly in the preference of the sex of their child.

## Results interviews

### Sample

Twenty-eight interviews were conducted: 15 men with male-related infertility and 13 women with unexplained, male- or female-related infertility. Only men diagnosed with male-related infertility participated in the interviews: This selection bias should be taken into consideration.

The men had a mean age of 35.9 years (range 29 – 45). The women had a mean age of 27.0 years (range 22 – 33). All participants were in different stages of infertility investigation. One woman had a child from her first husband. One man had an adopted child. The other participants were childless. All participants were married and living in the South of Vietnam.

### Motives for wanting children

Insight in the motives for wanting children is needed to understand the socio-cultural and psychological effects of infertility on couple's life in Vietnamese society. Both men and women think childbearing is the normal, expected thing to do after marriage. Answers to the question typically included: "You should at least have one child, every family has children", "The tradition is that marriage will lead to having a family", "Like other couples I want children", "You have to get children after marriage" and "Every married couple is supposed to have children". After further exploring the reason for their childwish they explained that children are very important for stabilizing their marriage and bringing happiness in their family lives, continuing the family line and for their elderly days. All eldest sons and daughters-in-law said: "It is important that I give my parents(-in-law) a grandchild to pass on our family name". They think children are a security for their old age in the way of financial support, caretaking and keeping company: "Children can take care of me when I am old" and "In the long future we might end up alone, that's why we need to have children".

### Psychological response/wellbeing

Some women expressed intense emotions when talking about their childless marriage and cried during the interview. Both men and women stated that they experienced feelings such as deep sadness, guilt, loneliness and fear for an insecure future. Family gatherings – like during the Tet-holidays-, children's birthday parties and the subject 'children' remind the interviewees of their childlessness and make them feel sad. The informants cope with these feelings in two different ways: avoidance and confrontation. Some participants, mainly women, stay at home avoiding social gatherings with children, while others go out to meet people or take care of nephews, nieces, foster-children or other children in the neighbourhood.

Being home makes men and women face the fact that they have no children. "The house feels empty without the sound of children" and "In the evening I imagine my whole house is filled with laughing, cheering and crying children. It feels like our house is too big for just the two of us", explained two men.

In order to forget their problems some men work very hard and try to keep themselves busy. A few men said the thought of their fertility problem affects their working skills in a negative way. Some men go out and abuse alcohol while their wives stay at home.

Nevertheless, all but one have a strong belief in the medical technologies and are very optimistic about their chance of having children in the future.

### Marital life

Worth knowing is that half of the participants were living with their parents(-in-law) during the time of the interviews.

Women with unexplained or female-related infertility felt their marriage was threatened and some were "afraid their husband would leave them". Two women with male-related infertility were joking: "My husband can take all women but it won't make a difference: He is the one with the fertility problem."

Many men stated that their wives were very sympathizing and supportive. Three infertile men offered their wives a divorce to give them a chance on motherhood. "I'm willing to divorce if she wants children from another husband." Most men and women wanted to solve the problem as a couple and even without children they will stay together. "We will be happy when we can have them, but without them we are still happy." One woman was acquainted with her husband's fertility problem before marriage; "He told me before the wedding so I could prepare. I was very surprised because I don't think other men will do the same."

Arguments between husband and wife about their childlessness are not uncommon. Both men and women tell that having a child would be the only way to stop the arguments. Remaining childless might lead to a divorce. In addition to verbal fights one woman with female-related infertility spoke about physical abuse: "During a fight my husband sometimes starts beating me, when he does, I never make a sound. After a fight he disappears for 2 or 3 days. (...) The only solution to stop the fights is to have a baby as soon as possible so that he will love our child and maybe there is a chance he will love me again."

### Secrecy and support

Women in particular were cautious and selective about whom they told their fertility problem. "We still use contraception" or "We're not ready yet for children" are lines commonly used by infertile men and women when people ask them why they don't have children. The majority is afraid of becoming the subject of gossips. Only three participants tell the truth: "the truth will come out someday" and "I'm afraid that people might think that it is my wife's problem, so I tell the truth". A few informants didn't want to speak at all about their fertility problem because "it is a private thing".

Partners are a source of support for most men and women. Families(-in-law) and friends who had been informed often gave advice about treatment and handling the secrecy, and supported them: "I think every member of our family should know so they can assist and we can share our feelings." Preventing stress, worries and pressure for/from their parents(-in-law) was the main reason for not telling them.

Thus the 'secrecy' with which many participants handled their childlessness appeared to be a barrier to support.

### Social pressure

Not having children after one year of marriage gives rise to questions such as "why don't you have children yet?" These confronting questions, although unintentionally, cause pain and feelings of pressure.

Mainly eldest sons and daughters-in-law are being pushed to have children as soon as possible to secure the lineage. The mothers-in-law in particular demand that the daughters-in-law will be examined and be treated unless there is proof for male infertility. Comments like "I'm relieved that my wife isn't the cause of our fertility problem, because now her relationship with my parents has been improved" and "When I come home from work my wife is often upset and tells me my parents, with whom we live, are pushing her to get pregnant" are evidence for the prejudice that women are supposed to be the cause of the infertility. Husbands needed to be persuaded by their wives to visit a doctor. Besides the 'negative' pressure, family-in-laws also suggests ways to conceive such as traditional food and medicine, and sources of western treatment. This advice may be encouraging to some couples, but may also bring too much pressure, stress and psychological harm. Many felt they should live up to the expectations of their family and were eager to please them.

One man and one woman could not live up to the expectations of their family and friends. "After three years of marriage without children we moved from our hometown to a place far away from our family and friends. We promised to return once we have children," said one man.

None of the informants felt isolated or treated differently because of the fact they do not have children. They were still invited to (children's) birthday parties, social gatherings and events by friends, colleagues and neighbours.

When asked which sex they favoured for their child, most answered: "My only wish is to have a healthy child of my own".

### Assisted reproduction, adoption and donor sperm

None of the participants had religious or cultural objections against assisted reproduction like IUI, IVF or ICSI. Adoption appeared not to be a favourite solution to meet their wishes for a family, because it would not be their own blood. The same applied to the use of donor-sperm, though some considered asking their brother(-in-law) as a donor.

### Economic consequences

The minority of the interviewees will undergo the necessary infertility treatment on their own expenses. However most of them need to work harder and/or take a loan in order to afford the treatments, others are forced to suspend due to insufficient finances. Parents (-in-law) who have a sufficient income often contribute to the payment of treatment.

The financial problems affect the couples' lives and cause distress. Interviewees mention the high costs of IVF treatment, "when IVF is necessary this will be the end for us because we can't afford it". Two participants started crying when their financial situation was brought up.

Some interviewees explained that their desire for children was so strong that they are 'willing to sell their house' in order to resolve their involuntary childlessness. One informant said: "I don't care about the amount of the costs as long as we are able to have children".

Many men and women are convinced that their financial future will depend on their children, without them they fear the future. In the same way they are taking care of their parents.

## Discussion

The findings of this study provide more insight in the consequences of infertility on couples' life in the South of Vietnam.

Most studies are unanimous in the conclusion that infertility is a more stressful experience for women than it is for men. A Study in Brazil [12], where the PET is a daily routine in assessing psychological distress, reported that the PET-score for women is significantly higher than for men (respectively 27 ± 8 and 22 ± 7). This study in the South of Vietnam suggests no significant difference between the PET-scores of husbands and wives. Remarkable is the difference in the PET-score between men in this study (27.38 ± 7.1) and the study in Brazil. The higher PET-score of men in the South of Vietnam can be interpreted by the higher values men in Vietnam have regarding their progeny: Children carry on the family name and are a consolidation for the future. Studies that found a difference between husbands' and wives' reaction on infertility were primarily conducted in developed modern western countries, while Vietnam is still a developing nation with an eastern culture.

The interviews showed the motives for wanting children for women are in-keeping with the findings of other studies in northern Vietnam [8-10]. Having children after marriage is in line with the Vietnamese tradition of the importance of family [13]. Children continue the family lineage and provide security for the future. The interviewed men did not differ in this from women. Assuming psychological consequences of infertility are affected by the motives for wanting children, it is not surprising that there is no difference between the PET-scores of husbands and wives.

Infertility causes a lot of grief, stress and sadness among men and women. All interviewees are reminded of their childlessness when confronted with seeing children or holding conversations about other children. No unambiguous conclusions about the participants' wellbeing can be made because duration and the cause of infertility, the stages of infertility investigation, the possibility of treatment and social- and economic background are all different for the participants. These factors influence the psychological responses [14].

In a study in Taiwan wives with a diagnosed female-related infertility experienced higher psychological distress than wives with a male-related infertility [7]. The results in this study show no difference between wives regardless of the diagnoses. The PET-scores between husbands with different diagnoses showed no significant difference either. The fact that the questionnaires were only filled in by couples who visited the hospitals together, might suggest that the couples consider the infertility as 'theirs' and not as a problem of 'one of them' individually. On the other hand, it is more in the line of expectations to think that women with female infertility would have a higher PET-score than women with other diagnoses because they might be blamed by or feel guilty to their family-in-law. Husbands with male infertility responded the same as the husbands with other infertility diagnoses. This corresponds with the findings among husband in Taiwan.

Almost one-third of the questionnaire participants have a PET-score that suggests they may need psychological support. This need was equal for husbands and wives regardless the cause of their infertility. Psychological support is not a part of the infertility treatment in the South of Vietnam.

In the interviews it was found that the way in which interviewees cope with their emotions due to their childlessness differs between men and women. Most women stay at home avoiding gatherings with children, while men rather leave the house, abuse alcohol and keep themselves busy in order to forget about their problem for a while. Gender differences in coping with infertility have been noticed in other studies [15,16]. The tendency of Vietnamese women to stay at home could partly be explained by the traditional thought that women are responsible for the household [9,17].

Studies from Africa and Asia report the subordinated role of women within their marriage. Women experiencing infertility felt their marriage was threatened and feared abandonment by their husband. [8,9,18]. Similarly in our study some women feared their husband would leave them. However women with male-related infertility felt themselves strengthened in their marriage. Surprisingly some men, who felt responsible for their childless marriage, were willing to divorce to give their wives a chance on motherhood. Interviewees wanted to solve the problem together. Yet, women were the first seeking medical help under coercion by in-laws who are convinced that women are the cause of childless marriages. The moment their test results were normal their husbands visited the doctor with or without persuasion of their spouses and integration into the family-in-law was regained. Contrary to studies in resource-poor countries that revealed a lack of male involvement in solving the infertility problem, even in cases of male factor infertility [19] husbands in this study were willing to solve their fertility problem and supported their wives during tests and treatment. As we only included couples instead of individuals, this outcome is not surprising – it may be expected that wives and husbands are both involved in the search for a solution for their fertility problem when they visit the hospital together.

In order to avoid people knowing about their infertility, nearly all of the interviewed men and women pretended that they did not wish to conceive yet. Similar tactics were found among infertile women in Bangladesh [20] and South-Africa [18]. The reason for this secrecy of the interviewees was their fear of becoming subject of gossips. On the other hand, this secrecy prevents them from getting support.

Pressure is brought to bear upon infertile couples by confronting questions about their childless marriage from people around them. Family interference is felt as a negative pressure and/or as an encouragement. Especially the eldest sons and daughters-in-law are pushed to give their parents grandchildren. It must be noted that eldest sons are supposed to co-habit with their parents [10], which can be the reason they feel more pressured. Almost fifty percent of the couples in our questionnaire lived with their parents. Studies in developing countries who have evaluated the social implications of infertility describe stigmatisation and social deprivation. This was not reported by couples in this study, even when specifically asked. It is possible that this is a result of the rapidly changing social and economic environment in Vietnam [8] and that in turn influences the flexibility with which people accept infertility. Besides this, the majority of infertile people uses a plausible excuse as to why they do not have children yet. Therefore people might not get the chance to stigmatize them.

Although very important in Vietnamese society [21,22] none of the informants mentioned a son-preference, their only wish is a healthy child. The 77.5 % with 'no preference' in the questionnaire support the latter.

Men and women would try anything in their power to see their childwish be fulfilled, though they do not consider adoption or the use of donor sperm. In the questionnaires only 18% considered adoption. Most interviewees believe any non-conjugal reproduction weakens the blood tie and leads to an unstable family, unless the brother-in-law impregnates the wife. This thought is widespread in Chinese-influenced Asia [3]. The statement 'I am against adoption because I only want a child of my own' shows once again the importance of the blood tie. On the other hand assisted reproduction is taken in consideration (67% of the questionnaire) but is often unaffordable. Couples have a strong belief in the (developments of) medical technology. However the treatment fees are quite high in relation to the average income: the cost of IVF-treatment is roughly USD 3.000 though almost half of the participants earns less than USD 140 per month. Consequently participants have to reduce their expenses and save all their money for infertility treatment. Family plays an important role by contributing to these costs. This economic hardship is a particular distressing factor for the infertile couple in the South of Vietnam. Bear in mind that all participants in this study could at least afford the costs of consulting a doctor. Without children people fear the future because they feel that they have a lack of social security and support in their old age.

Studies in developing countries show alcohol- and physical-abuse are common problems within a childless marriage. This study reports a few cases of men abusing alcohol. One woman has become victim of domestic violence; remarkably almost a similar signal has been reported by others [18]. Nevertheless physicians need to take these potentially dangerous problems into account. Enlarging the sample size will give a better insight in the prevalence of physical and alcohol abuse among infertile couples.

The response rate is unknown because the number of distributed questionnaires over the period of the study was not monitored. This is important to mention because there is no information about patients who did not want to participate or did not complete the questionnaire. Therefore it is unknown if the sample is representative for the population that visits the two clinics, although this method of data collection is convenient in busy fertility clinics.

The fact that almost half of the couples recognise their fertility problem within two years of marriage shows the importance for Vietnamese couples of having children in the future.

In conclusion this study shows that family-influences play an important role in the experiences of infertility, that the economic consequences on couples' lives should not be underestimated and that there is a need for psychological consultation and mental support in the program of infertility management in developing countries like, in this study, the South of Vietnam.

## Competing interests

The author(s) declare that they have no competing interests.
